# Motion sickness susceptibility modulates the impact of electrical vestibular stimulation on postural control

**DOI:** 10.1038/s41598-025-12683-3

**Published:** 2025-07-25

**Authors:** Karina Moïn-Darbari, Daniel Paromov, Benoit-Antoine Bacon, Maxime Maheu, François Champoux

**Affiliations:** 1 École d’orthophonie et d’audiologie, Université de Montréal, Montreal, H3N1X7 QC Canada; 2 Centre de recherche de l’Institut universitaire de gériatrie de Montréal (CRIUGM), Montreal, QC Canada; 3 Department of Psychology, The University of British Columbia, Vancouver, BC Canada

**Keywords:** Motion sickness, Electrical vestibular stimulation, Postural control, Multisensory integration, Neurophysiology, Auditory system, Motor control, Sensorimotor processing, Sensory processing

## Abstract

Motion sickness is defined as a sensation of uneasiness that occurs during physical motion, such as transportation by bus, plane, car or train. Postural control is one of the multisensory processes that has been examined in individuals susceptible to motion sickness. Indeed, postural control relies on visual, somatosensory and vestibular information. While studies suggest normal-like postural control during quiet standing, others suggest that individuals with motion sickness show increased postural instability during sensory perturbations, namely during visual and somatosensory disturbances. The impact of vestibular stimulation on postural control in individuals with motion sickness has yet to be determined. Therefore, the aim of the present exploratory study was to examine the effects of sinusoidal electrical vestibular stimulation on postural control in individuals with varying degrees of motion sickness. Fifty participants were divided into three groups based their susceptibility to motion sickness. Participants were initially tested at baseline in the four postural conditions of the modified Clinical Test of Sensory Integration and Balance protocol (mCTSIB): eyes open on firm surface, eyes closed on firm surface, eyes open on foam surface, eyes closed on foam surface. These four conditions were then repeated during sinusoidal electrical vestibular stimulation (EVS) of 1 mA at 1 Hz. In baseline conditions, no significant group differences in postural control were found. Data in experimental (EVS) conditions, however, suggests that individuals with high susceptibility to motion sickness are more impacted by vestibular stimulation, specifically in the eyes closed on firm surface condition. It has been suggested that motion sickness could be the result of an altered multisensory integration process. While the present data do not allow us to answer this question, it would remain important to examine all types of sensory perturbations and combinations thereof in a larger group of individuals.

## Introduction

Motion sickness is defined as a sensation of uneasiness that occurs during physical motion of the individual, such as during transportation by bus, plane, car or train. It can extend from a vague sense of discomfort up to nausea and vomiting^1^. Possible symptoms include, to varying degrees of severity, nausea, sweating and pallor, sopite syndrome (drowsiness), vertigo and headache. These symptoms must appear during motion and build with prolonged exposure and cease after motion has ended^2^. Motion sickness affects 25–30% of adults, to varying degrees ranging from mild to severe motion sickness^3–5^. The currently available treatments are limited and invasive. Medications such as antimuscarinic, antihistamine and sympathomimetic agents pose the risk of side effects such as fatigue and the risk of dependence^6^. Behavioral treatments by habituation require several sessions before taking effect, cause significant discomfort for the individual, and are stimulus-dependant^7–10^. This limitation in available treatments could be due to a poor understanding of the mechanisms behind motion sickness.

Postural control is one of the multisensory processes that has been examined in individuals susceptible to motion sickness. Postural control is a rapid and effective method to study sensory interactions by modulating the availability of various sensory cues^11–15^. Previous studies have aimed at demonstrating the specific influence of vision or somatosensory cues on postural control in individuals susceptible to motion sickness. Indeed, they have shown that individuals with motion sickness do not differ from control groups in postural control during a simple standing task without any sensory perturbation^16,17^. On the other hand, during visual and somatosensory disturbances, certain differences seem to emerge. Results suggest that visual or somatosensory disturbances have a greater impact on the surface area and velocity of the center of pressure (CoP) in individuals susceptible to motion sickness compared to control participants^18–20^. This suggests that these individuals do not process sensory information for postural control in the same way as individuals without motion sickness during sensory disturbances. The impact of vestibular stimulation on a postural control task in individuals with motion sickness has yet to be determined. This information would be important to determine whether the performance impairments noted during visual and somatosensory perturbations are generalized to all the senses or only impacting specific sensory systems.

Vestibular perturbation can easily be induced with electrical vestibular stimulation (EVS). Such stimulation generates a small current delivered through the mastoids, which is known to disrupt peripheral vestibular signals of the area encompassing the semicircular canals and the otoliths^21–23^. The aim of the present study was to examine the effects of EVS on postural control in individuals with varying degrees of motion sickness.

## Methods

The study was approved by the ethics committee of the Université de Montréal (CERC-2023-4023). All methods were performed in accordance with the relevant guidelines and regulations. Informed written consent was obtained from all participants. Research was performed in accordance with the Declaration of Helsinki.

### Participants

Fifty individuals participated in the study. Participants were healthy and did not report any history of head trauma, dizziness/vertigo or uncorrected vision impairment. All participants had normal hearing as verified by 250–8000 Hz pure tone audiometry by a qualified audiologist, as well as normal horizontal semicircular canal function as verified by video Head Impulse Test (ICS Impulse, Otometrics, Denmark).

Participants initially filled out the Motion Sickness Susceptibility Questionnaire – Short version (MSSQ - Short)^24,25^. Participants were then divided into three groups: low, moderate and high susceptibility to motion sickness. Those scoring equal to or above the 75th percentile classified as having a high susceptibility to motion sickness, those scoring equal to or below the 25th percentile (raw score = 5) as having a low susceptibility to motion sickness, and those between the 25th and 75th percentile as having moderate susceptibility to motion sickness^24^.

### **Procedure**

Participants were assessed with the modified Clinical Test of Sensory Interaction and Balance protocol (mCTSIB) on a force platform (Accusway, AMTI, USA) in a single session. The mCTSIB measures postural control in conditions with eyes open or closed, and on a firm or foam surface, to isolate the different sensory components (vision, somatosensory) that are necessary to maintain balance^26^. The mCTSIB has been widely used as a reliable clinical tool, validated in healthy adults^27–29^and has also already been used in healthy adolescents with motion sickness^16^. The sampling rate of the platform was set at 100 Hz and each trial lasted 30 s, providing 3000 samples^30^. Participants were asked to remove their shoes and stand in an upright position, with their feet positioned shoulder width apart and arms at their side. Once the 30 s trial was over, participants were asked to step down from the platform and given a 15 s rest before the next trial. Participants stood in four different postural conditions: Eyes open on a firm surface, Eyes closed on a firm surface, Eyes open on foam (AIB Balance Foam, AIB, USA), Eyes closed on foam. For the conditions with eyes open, participants were asked to look at a target at eye level on a wall, 1.5 m away. Each sensory condition was repeated three times in the recommended predetermined order^26^ and the median value of each condition was retained for each participant. If participants failed one of the three trials, the median of the successful two remaining trials was retained. If participants failed to complete more than one of three trials in a condition, their data was excluded from analysis for that specific condition.

Following baseline measures, EVS was delivered through a bench-top electrical vestibular stimulator (Soterix Medical, USA). Round-shaped electrodes of 3 cm^2 ^were placed bilaterally over the mastoids. Bipolar sinusoidal current of 1 Hz was applied. To ensure experimental validity and control for individual differences in vestibular sensitivity, each participant’s vestibular perception threshold to EVS was first determined. The vestibular perception threshold refers to the minimum intensity of vestibular stimulation—here, specifically from EVS—at which an individual consciously perceives a vestibular sensation, such as a sense of movement, tilt, or sway. In protocols where the experimental EVS current is fixed across subjects, an individual with a lower vestibular threshold could experience increased effects of EVS compared to an individual with a higher threshold, therefore skewing the results^31^. As such, individual thresholds were measured carefully to ensure no statistical differences between groups could be induced by a lower threshold to GVS in the high susceptibility group. Current was slowly increased from 0 mA by an average of 0.2 mA/sec. Participants were asked to verbally say ‘yes’ when they started perceiving the induced postural sway. Participants were also asked to refrain from saying ‘yes’ when only perceiving a tingling sensation but were told to expect a sensation like being on a boat and report when this sensation started. This vestibular perception threshold search was done 3 times and the median of the three trials was retained for each participant. Following the threshold search, the same four postural conditions assessed in baseline were repeated, but this time while EVS was delivered at a stimulation intensity of 1 mA.

Raw CoP data were exported into Matlab (R2022b) and parsed through a custom-made script to retrieve variables of interest (sway area of the 95% confidence ellipse, total sway velocity antero-posterior sway path length and velocity as well as mediolateral sway path length and velocity) for each postural control condition during both baseline and stimulation measurements. Variable distribution was verified prior to selecting an appropriate statistical method of analysis. Shapiro-Wilk tests confirmed that data were not normally distributed for sway area in all conditions tested (*p* <.001) as well as for sway velocity during EVS conditions (*p* <.001 to *p* =.03). Additionally, data were also not normally distributed for mediolateral sway path in all conditions tested (*p* <.001) as well as for anteroposterior sway path during EVS conditions (*p* <.03). Finally, data were not normally distributed in all anteroposterior (*p* <.001 to *p* =.048) and mediolateral (*p* <.001) sway velocity conditions. Therefore, Kruskal-Wallis non-parametric analyses were performed to examine between-group differences on 95% confidence ellipse of sway area and total sway velocity, as well as antero-posterior and mediolateral sway path length and velocity.

## Results

In accordance with the Motion Sickness Susceptibility Questionnaire scores, 14 participants (12 women) were included in the low susceptibility to motion sickness group (mean score: 2.20 ± 1.85), 18 participants (15 women) in the moderate susceptibility group (mean score: 9.64 ± 3.33) and 18 participants (all women) were classified as having a high susceptibility to motion sickness (mean score: 28.90 ± 7.25). No significant differences between groups were reported for age (group 1: 27.93 ± 0.363; group 2: 26.17 ± 7.66; group 3: 24.94 ± 3.56), height (group 1: 1.66 ± 0.10 cm; group 2: 1.64 ± 0.07 cm; group 3: 1.62 ± 0.05 cm) or weight (group 1: 143 ± 29 lbs; 2: 135 ± 21 lbs; group 3: 135 ± 24 lbs). Groups were balanced in terms of handedness (group 1 and 2: 1 left-handed; group 3: 2 left-handed). All participants were able to successfully complete all three trials in baseline conditions. However, during the fourth EVS condition (Eyes closed on foam), several participants (4 participants in group 1, 2 participants in group 2 and 4 participants in group 3) failed to successfully maintain posture for the duration of the trial and therefore were not able to complete more than one trial. Their data was therefore excluded from analysis for this condition.

In the baseline condition, there were no significant differences between groups across postural control conditions for either sway area (Condition 1: χ^2 ^(2) = 2.190, *p* =.335; Condition 2: χ^2 ^(2) = 0.756, *p* =.685; Condition 3: χ^2 ^(2) = 0.197, *p* =.906; Condition 4: χ^2 ^(2) = 0.261, *p* =.878) or sway velocity (Condition 1: χ^2 ^(2) = 0.352, *p* =.839; Condition 2: χ^2 ^(2) = 0.661, *p* =.719; Condition 3: χ^2 ^(2) = 2.799, *p* =.247: Condition 4: χ^2 ^(2) = 1.959, *p* =.376).

**Fig. 1:**
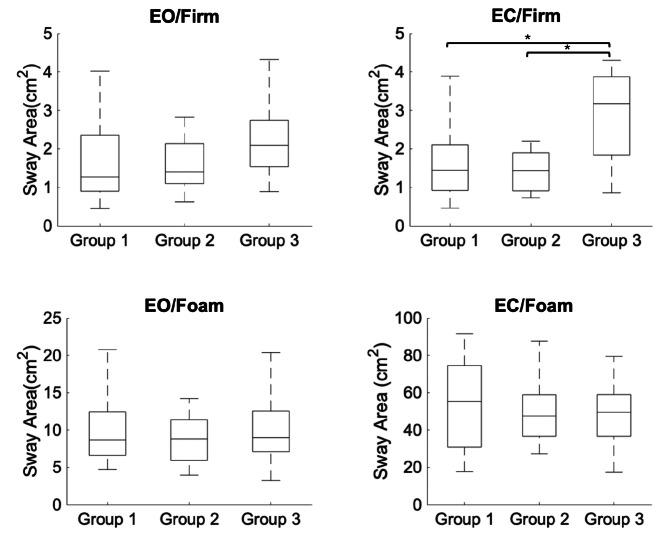
Average sway area in the four postural control conditions (Eyes open on firm surface; Eyes closed on firm surface; Eyes open on foam; Eyes closed on foam) for the three groups (group 1: low susceptibility; group 2: moderate susceptibility; group 2: high susceptibility to motion sickness) during electrical vestibular stimulation (EVS). On each box, the central mark indicates the median, and the bottom and top edges of the box indicate the 25th and 75th percentiles, respectively. Error bars represent the minimum and maximum value that are not considered outliers. *p <.05.

During EVS, however, there was a significant between-group difference in the second postural condition (Eyes closed on a firm surface) in both sway area (χ^2 ^(2) = 10.430; *p* =.005) and sway velocity (χ^2 ^(2) = 8.192; *p* =.017). Post hoc Dunn-Bonferroni tests revealed that individuals who were highly susceptible to motion sickness showed an increased sway area in this condition during GVS as compared to both moderately susceptible (*p* =.017) and less susceptible (*p* =.017) groups (Fig. 1).

**Fig. 2:**
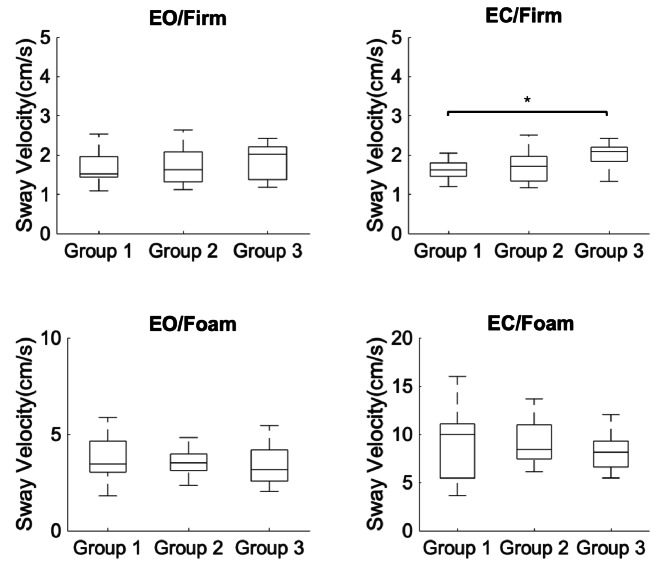
Average sway velocity in the four postural control conditions (Eyes open on firm surface; Eyes closed on firm surface; Eyes open on foam; Eyes closed on foam) for the three groups (group 1: low susceptibility; group 2: moderate susceptibility; group 2: high susceptibility to motion sickness) during electrical vestibular stimulation (EVS). On each box, the central mark indicates the median, and the bottom and top edges of the box indicate the 25th and 75th percentiles, respectively. Error bars represent the minimum and maximum value that are not considered outliers. *p <.05.

In addition, individuals with high susceptibility to motion sickness showed increased sway velocity in the second condition when compared to the group with low susceptibility to motion sickness (*p* =.033) but failed to demonstrate a significant difference with the moderate susceptibility group (*p* =.057) (Fig. 2). No other significant differences in sway area were found during GVS for postural conditions A (χ^2 ^(2) = 4.947, *p* =.084), C (χ^2 ^(2) = 0.452, *p* =.798) or D: χ^2 ^(2) = 0.093, *p* =.954, nor for sway velocity in conditions A (χ^2 ^(2) = 2.831, *p* =.243), C (χ^2 ^(2) = 0.867, *p* =.648) or D (χ^2 ^(2) = 0.877, *p* =.643). In all measurements, there were no statistical differences bewtwwen groups 1 and 2 (low and moderate susceptibility) and no significant group differences (*p* <.05) were found in any of the baseline or EVS anteroposterior or mediolateral sway path length or sway velocity components.

Finally, no significant differences were found in vestibular perception threshold between groups (group 1 mean: 0.344 ± 0.124 mA, group 2: 0.381 ± 0.226, group 3: 0.320 ± 0.160, χ^2 ^(2) = 0.881, *p* =.644). This confirms that the statistical differences between groups are not a consequence of a lower threshold in the highly susceptible group.

## Discussion

The aim of the present study was to examine the effects of EVS on postural control in individuals with various degree of motion sickness. The present study suggests that individuals with a high susceptibility to motion sickness do not differ from individuals with low susceptibility to motion sickness in all baseline conditions. These results are consistent with previous studies showing normal postural control in individuals with variable degrees of susceptibility to motion sickness^16,17^. During EVS, however, individuals with high susceptibility exhibit increased postural instability, as defined by a marked increase in sway area and sway velocity. More specifically, such instability was found when visual cues were absent, suggesting that the remaining congruent sensory information, namely somatosensory inputs, are not sufficient to resolve the sensory conflict. Previous studies show that individuals with higher susceptibility to motion sickness exhibit more postural instability during sinusoidal visual perturbations^32^ and during somatosensory perturbations such as calf muscle vibration^18^. The present study adds to the litterature on sensory perturbations by suggesting that these individuals also exhibit poorer performance during vestibular stimulation. Therefore, this increased instability in postural control is possibly generalized to all sensory perturbations. Considering the increase in sway in motion sick individuals, one may have expected a greater number of falls in group 3. However, falls occurred relatively similarly across groups, and only during the most challenging condition, namely in the fourth condition (eyes closed/foam surface) during EVS. The difficulty of the task has been shown to produce great variability in postural control performance^29^. Such variability in performance may not reliably discriminate the impact of EVS between groups. Finally, the mCTSIB protocol, as standardized by Cohen et al. (1993), specifies a fixed order for the four conditions (eyes open/firm, eyes closed/firm, eyes open/foam, eyes closed/foam) to ensure consistency and reliability. As such, we followed this protocol to maintain clinical validity. The fixed mCTSIB order may arguably introduce minor order effects, though rest periods and participant preparation for EVS sensations likely minimized these. Future studies could counterbalance conditions or assess confidence levels to further control for this possibility.

In line with the sensory conflict theory, the poorer postural control performance exhibited in motion sick individuals could emerge from a discordance between visual, vestibular and somatosensory information and the movements predicted in the internal model^33–36^. The sensory conflict theory, however, does not make any predictions about sway in the present experiment. In this study, no one became sick. The sensory conflict theory would argue that no one became sick because there was no sensory conflict. This question is important, in part, because other theories of motion sickness etiology make predictions about differences in postural sway between susceptible and less susceptible groups prior to exposure to potentially nauseogenic stimuli. If the sensory conflict theory is the “most common hypothesis”, alternative theories of motion sickness etiology should be considered (for a recent review of theories, see^37^). The ecological hypothesis (or postural instability hypothesis), for example, makes an explicit prediction that sway should differ between low and highly susceptible participants, and that such differences should exist before the onset of any symptoms of motion sickness^38,39^. This prediction has been confirmed in many settings and groups^37^. Although the aim of the present research was not to confirm either of these theories, it appears to be consistent with the latter.

Several challenges stand in the way of studying the many theories underlying motion sickness. One of the major challenges in studies looking at individuals with motion sickness is the categorization of severity of motion sickness. Questionnaires are the currently the only tools available to assess individuals’ symptoms. The MSSQ does not collect severity of motion sickness, or time to onset of symptoms, nor specific situations (reading in the car, passenger in front or back seat, avoidance of certain modes of transportation known to elicit sympotoms, etc.). More objective methods in evaluating motion sickness should eventually be developped in order to increase specificity and sensitivity of varying degrees of motion sickness. These methods would be necessary in order to present definitive indicator of motion sickess suceptibility. Notably, postural kinematics could be a promising avenue to develop objective measures of susceptibility^40^. Additionally, it could also be noted that participants were not screened for migraine, which is linked to motion sickness and may affect sensory integration^41,42^nor for anxiety, though it can also influence postural control and motion sickness susceptibility^25,43^. Future studies should include migraine screening and should incorporate validated anxiety questionnaires, such as the HADS, to control for these variables. Gender is also a carasterictic of motion sickness that may needs to be explored further. Indeed, our data corroborate the notion in which motion sickness is more prevalent in women^44^. On a fundamental point of view, one may wonder why men seem more resistant to motion sickness. On a more methodological point of view, this variable should be more carefully controlled in futur studies in order to eliminate any possible gender effects. Finally, the types of motion sickness should be the subject of comparative studies. In the present study, participants were recruited in relation to motion sickness linked to modes of transportation. It is plausible, however, that different processes underlie different types of motion sickness, such as visually induced motion sickness and cybersickness. The study of these distinct issues could allow us to better understand whether the mechanisms underlying each of these disorders are similar or distinct. It is likely that the different theories are not incompatible, but linked more strongly to one type of motion sickness rather than another, while concomittanly having distinct neural substrates underlying these conditions. Finally, it has been suggested that motion sickness could be the result of an altered multisensory integration process. While the present data do not allow us to answer this question, it would remain important to examine all types of sensory perturbations and combinations thereof in a larger group of individuals.

## Data Availability

The datasets generated and analysed during the current study are available from the corresponding author on reasonable request.
